# The Relationship between Perivertebral Venous Cement Embolism and Balloon Expansion Pressure in Balloon Kyphoplasty

**DOI:** 10.31662/jmaj.2021-0065

**Published:** 2021-09-01

**Authors:** Yusuke Tabata, Shuhei Matsui, Masabumi Miyamoto, Takao Nakajima, Tokifumi Majima

**Affiliations:** 1 Department of Orthopedic Surgery, Nippon Medical School, Tokyo, Japan; 2 Department of Orthopedic Surgery, Musashino General Hospital, Saitama, Japan; 3 Department of Orthopedic Surgery, Nippon Medical School Chiba Hokusoh Hospital, Chiba, Japan; 4 Department of Orthopaedic Surgery, Sekishindo Hospital, Saitama, Japan

**Keywords:** Osteoporotic vertebral compression fractures (OVCFs), balloon kyphoplasty (BKP), cement embolization, balloon expansion pressure (BEP), perivertebral veins

## Abstract

**Introduction:**

Osteoporotic vertebral compression fractures (OVCFs) are common fractures in the elderly suffering osteoporosis. Most patients have bone fusion with deformity of vertebral collapse; however, some patients suffer nonunion and persistent pain at the fracture site. Due to the limitations of conservative treatment, balloon kyphoplasty (BKP) has been recently performed for OVCFs. This study aimed to investigate the relationship between cement embolization and balloon expansion pressure (BEP) in patients who underwent BKP.

**Methods:**

We investigated 62 patients who underwent BKP for cement embolization into the perivertebral veins among the 155 patients admitted to our hospital due to thoracolumbar vertebral compression fractures between April 1, 2019, and March 31, 2020. Surgery was indicated for patients who had severe back or low back pain and whose daily life was severely impaired, and in whom the shape of the vertebral body was clearly changed on functional X-ray.

**Results:**

Intraoperative X-ray and postoperative CT revealed cement embolization into the perivertebral veins in three cases (4.83%). The BEP was significantly higher in the group with cement embolism than in the group without cement embolism (P < 0.001). Pulmonary cement embolism (PCE) and infection were not observed. One case of cement leakage into the spinal canal was observed (1.61%).

**Conclusions:**

While the surgical intervention of BKP can contribute to the treatment of OVCFs, careful attention should be paid to the prevention of complications, including cement embolization into the perivertebral veins, and such complications should be appropriately managed.

## Introduction

Osteoporotic vertebral compression fractures (OVCFs) are common fractures in the elderly suffering osteoporosis. Fujiwara et al. reported that the incidence of vertebral compression fractures was approximately twice as high in women as in men and gradually increased with age ^(1)^. It was reported that the relative risk of incidence of vertebral fractures increased with the number of existing previous vertebral fractures (3.2-fold for one fracture, 9.8-fold for two fractures) ^(2)^, leading to a domino effect after the first vertebral compression fracture. Kado et al. demonstrated that women with one or more vertebral fractures had 1.23-fold higher age-adjusted mortality rate than women who had no vertebral fractures ^(3)^. OVCFs impair activities of daily life in the elderly, and we need to urgently deal with their prevention and treatment.

Fundamentally, OVCF patients are initially treated conservatively using a brace and rehabilitation. Most patients have bone fusion with deformity of vertebral collapse; however, some patients suffer nonunion and persistent pain at the fracture site. Moreover, there is a possibility that bone fragments may protrude into the spinal canal and cause spinal cord injury. Tsujio et al. reported that 13.5% of 350 patients had nonunion after conventional conservative treatments for OVCF in a prospective multicenter study ^(4)^. More recently, Inose et al. reported that 17.5% of 166 OVCF patients had nonunion in another prospective cohort study ^(5)^. Noteworthy findings that can easily lead to nonunion include cleft within the vertebral body, thoracolumbar fractures, high-intensity localized change on T2-weighted image of magnetic resonance imaging (MRI), wide range of low-intensity change on T1-weighted MRI, and fractures of the posterior vertebral body wall ^(5), (6)^.

Due to the limitations of conservative treatment, surgery has been recently performed for OVCFs that are refractory and have delayed bone fusion. The first percutaneous vertebroplasty (PVP) was performed in 1987 for vertebral angiomas ^(7)^ and has since been widely used in the treatment of OVCFs ^(8)^. While PVP can be rather easily performed under local anesthesia, the application of high pressure and injection of low-viscosity cements in PVP sometimes lead to cement embolization in the blood and leakage of cement into the vertebral canal ^(9), (10)^. Since PVP is mainly intended for pain relief and often reduces fractures inadequately, balloon kyphoplasty (BKP) was developed to expand a balloon to reduce the fracture and then fill in the resulting cavity after balloon removal with bone cement ^(11)^. Because a cavity has been formed by using a balloon, it becomes possible to inject bone cement with low pressure and high consistency. As a result, it was reported that the rate of cement leakage into the spinal canal with BKP was lower than that with vertebroplasty ^(12), (13), (14)^.

To our knowledge, there have been no detailed reports of investigations into the relationship between cement embolization of blood vessels and balloon expansion pressure (BEP) in BKP. In this study, we investigated the rate of cement embolization into the perivertebral veins, the relationship between cement embolization of blood vessels and BEP, and other complications of BKP.

## Materials and Methods

We investigated 62 patients who underwent BKP for cement embolization into the perivertebral veins among the 155 patients admitted to our hospital due to thoracolumbar vertebral compression fractures between April 1, 2019, and March 31, 2020. The indications for BKP were as follows: patients who had severe back or low back pain and whose daily life was severely impaired and in whom the shape of the vertebral body was clearly changed on functional X-ray. All the patients, except for two who were not able to undergo MRI due to aneurysmal clipping or pacemaker, underwent functional X-ray, computed tomography (CT), and MRI. Functional X-ray was performed by comparing the lateral images of sitting flexion position and supine extension position. Three orthopedic surgeons and one radiologist interpreted all images.

BKP was performed under general anesthesia using biplane imaging techniques. It was performed with access to both pedicles. The goal was to inject as much bone cement as possible to regain the height of the vertebrae up to that of the supine position seen in X-ray before surgery.

The temperature in the operation room was kept constant at 25°. Bone cement was kept refrigerated before surgery and always used at a constant viscosity in surgery.

Patients who underwent BKP were evaluated for whether they developed pulmonary cement embolism (PCE) by respiratory condition and D-dimer level in the blood.

The research was approved by the IRB of Musashino General Hospital, and the approval code issued by the IRB was No.11.

## Results

**Figure 1.**
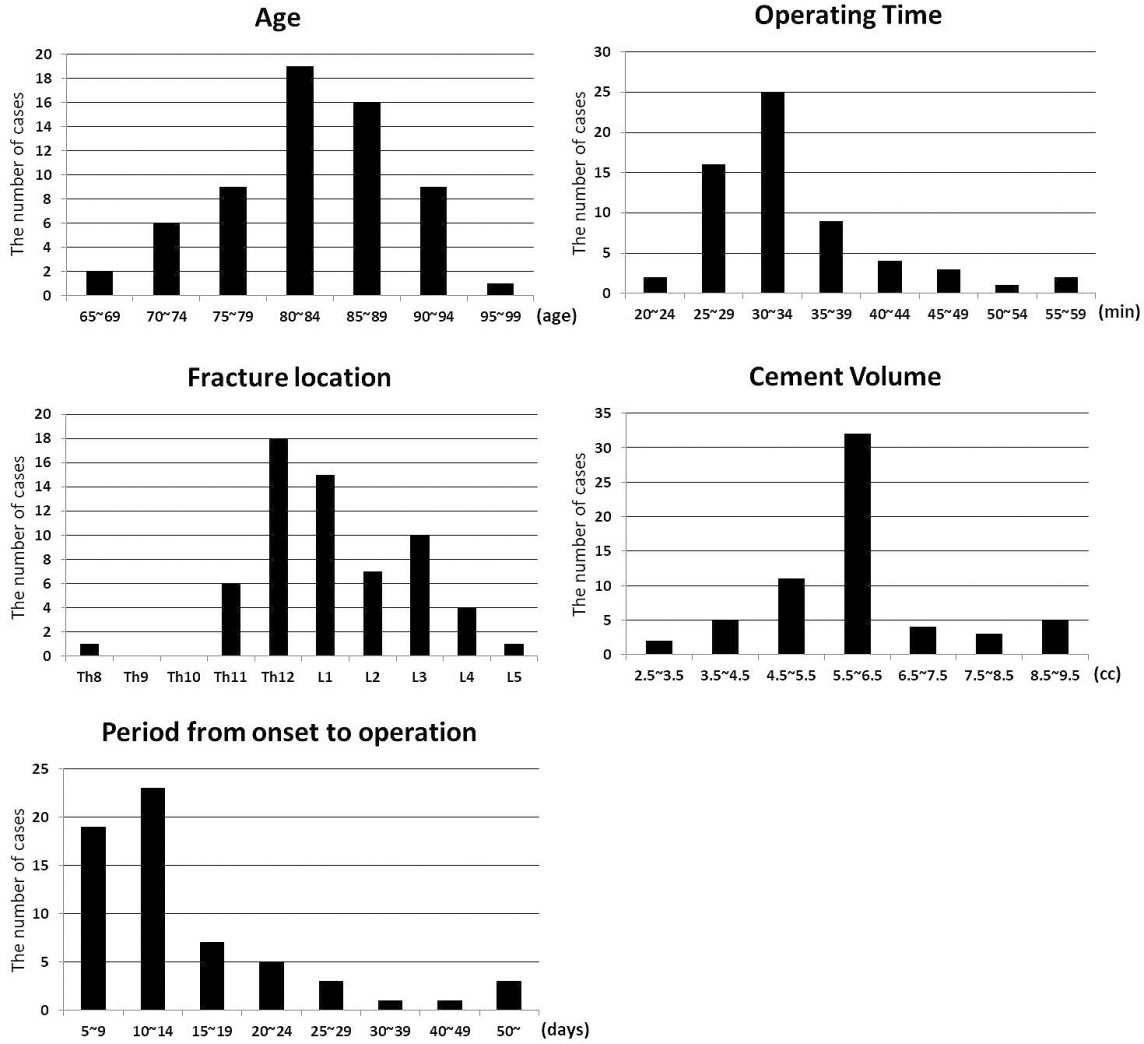
Distribution of age, operating time, fracture location, cement volume, and period from onset to operation.

BKP was performed in a total of 62 patients in this study. Surgery was performed on a single vertebral body. The mean age of the patients was 82.7 (±SD 6.7) years, and 66.1% of them were women. In the distribution of fracture regions, most fractures occurred at Th12. The mean operating time was 33.9 min (±SD 7.3). The average cement volume used in operation was 5.9 mL (±SD 1.4), and the period from onset to operation was 16.1 days (±SD 14.7) (Figure 1).

Intraoperative X-ray and postoperative CT revealed cement embolization into the perivertebral veins in three cases (4.83%). The demographic data of the two patient groups (with cement embolism and without cement embolism) are summarized in Table 1. While there was no statistically significant difference in sex, age, operation time, cement volume, and period from onset to operation between the group with cement embolism and that without cement embolism, the BEP was significantly higher in the group with cement embolism than that without cement embolism (P < 0.001) (Table 1). Pulmonary cement embolism and infection were not observed. One case of cement leakage into the spinal canal was observed (1.61%).

**Table 1.** Patient Demographic Data (Two-tailed Student’s t-test).

	Cement embolisms (3 patients)	No cement embolisms (59 patients)	P values
Male/Female	1/2	20/39	0.984
Age	77.3 (±SD 4.0)	83.0 (±SD 6.7)	0.156
Operating Time (min)	33.0 (±SD 5.3)	34.0 (±SD 7.5)	0.832
Cement volume (ml)	5.0 (±SD 1.7)	6.0 (±SD 1.3)	0.242
Period from onset to operation (days)	9.3 (±SD 3.1)	16.4 (±SD 15.0)	0.417
BEP (psi)	161.0 (±SD 16.5)	103.1 (±SD 19.0)	<0.001

### Case 1

**Figure 2.**
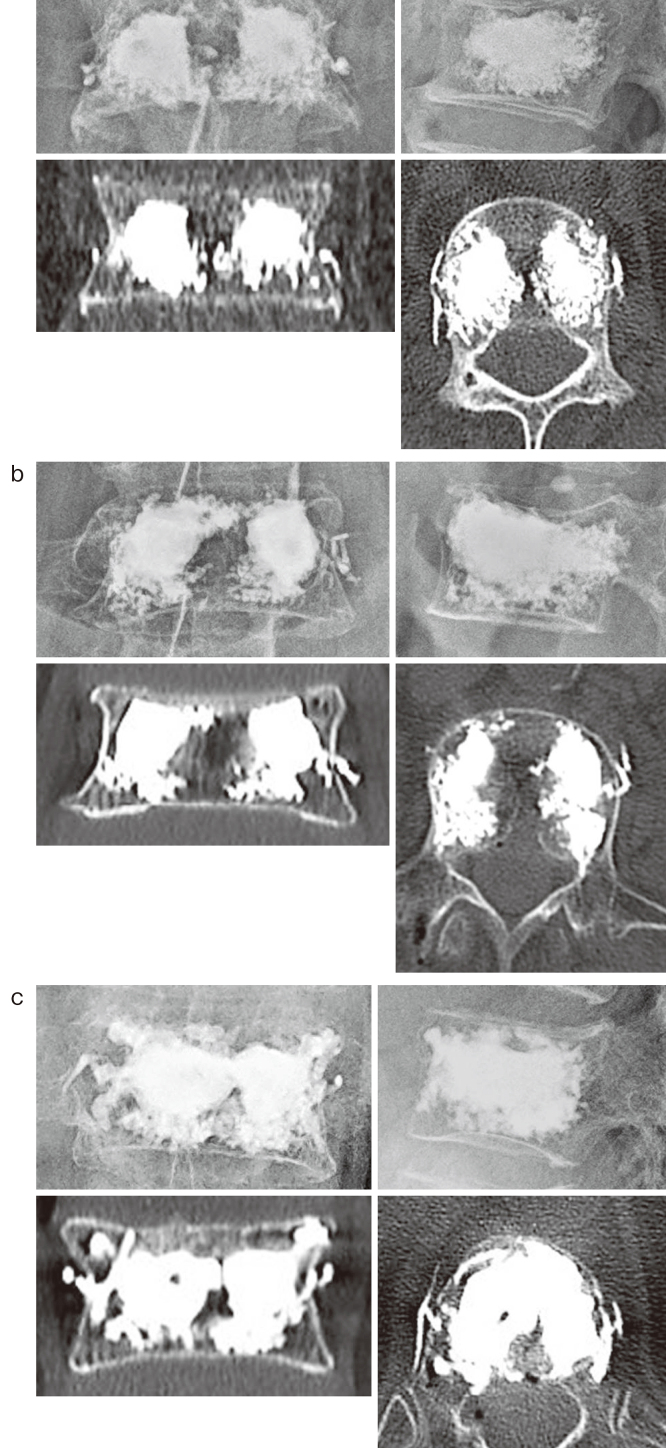
Postoperative X-rays from three cases who had cement embolization into the perivertebral veins. (a) Case 1. (b) Case 2. (c) Case 3.

An 81-year-old man fell from his chair in his home and could not walk due to low back pain; thus, he was brought to our hospital by ambulance. X-ray, CT, and MRI showed an L1 compression fracture, and he was admitted as an emergency. BKP was performed 12 days after the onset. The operation was completed in 39 min using 6 mL of bone cement (Medtronic, Ireland) without any issues. The BEP was high, approximately 160 psi. Intraoperative X-ray and postoperative CT revealed cement embolisms in both the right- and left-side veins of the L1 vertebral body (Figure 2a). There was no sign of PCE. His pain was immediately reduced after surgery, he was allowed to walk the next day with a brace, and he was discharged from our hospital.

### Case 2

A 78-year-old woman fell in her home and developed low back pain. She became unable to stand 2 days later. She was brought to our hospital by ambulance and admitted as an emergency. X-ray, CT, and MRI demonstrated an L3 compression fracture, and BKP was performed 10 days after the onset. While the operation finished in 31 min using 6 mL of bone cement without issue, the BEP was approximately 180 psi. Intraoperative X-ray and postoperative CT revealed a cement embolism in the left-side vein of the L3 vertebral body (Figure 2b), and no PCE occurred. Her pain was immediately improved after surgery, and she was discharged from our hospital.

### Case 3

A 73-year-old woman experienced back pain after lifting heavy weights. She became unable to walk on the same day, and she was brought to our hospital by ambulance. X-ray, CT, and MRI showed a Th12 compression fracture, and she was admitted as an emergency. BKP was performed 6 days after the onset. The operation finished in 29 min using 3 mL of bone cement without issue; however, the BEP was approximately 145 psi. Intraoperative X-ray and postoperative CT revealed cement embolism in both the right- and left-side veins of the Th12 vertebral body (Figure 2c). There was no sign of PCE. Her pain was immediately reduced after surgery, and she was discharged from our hospital.

## Discussion

It has been reported that BKP has an analgesic effect immediately after surgery because the cement injected into the vertebral body hardens in a short time and decreases the mechanical stress associated with activity. It has also a significant effect on the prevention of physical weakness and dementia by enabling patients to leave the bed at an early stage ^(15), (16)^. Moreover, it has been reported that cytotoxic and exothermal action destroys the nerve endings of the bone, which also helps reduce pain during cement polymerization ^(17)^.

In 2009, two papers questioning the effectiveness of PVP for OVCFs were published ^(18), (19)^. However, thereafter, the results of randomized controlled trials and open-label randomized studies were reported successively, demonstrating the efficacy and safety of BKP and PVP ^(20), (21), (22), (23)^. Currently, the efficacy of BKP is recognized worldwide, and it has been performed in many OVCF patients. While BKP is a low-invasive surgical treatment and can be performed even in the elderly, it also presents risks of complications, including infection, PCE, and cement leakage into the spinal canal. In this study, a remarkable analgesic effect was observed in all patients, and rehabilitation could be started earlier than conservative treatment. Although cement leakage into the spinal canal occurred in one case, the amount of leakage was small, and no neurological symptoms were observed. Cement embolism into the perivertebral vein occurred in three cases (4.83%); however, no physical abnormalities were observed, including PCE; thus, no additional treatment was required.

**Figure 3.**
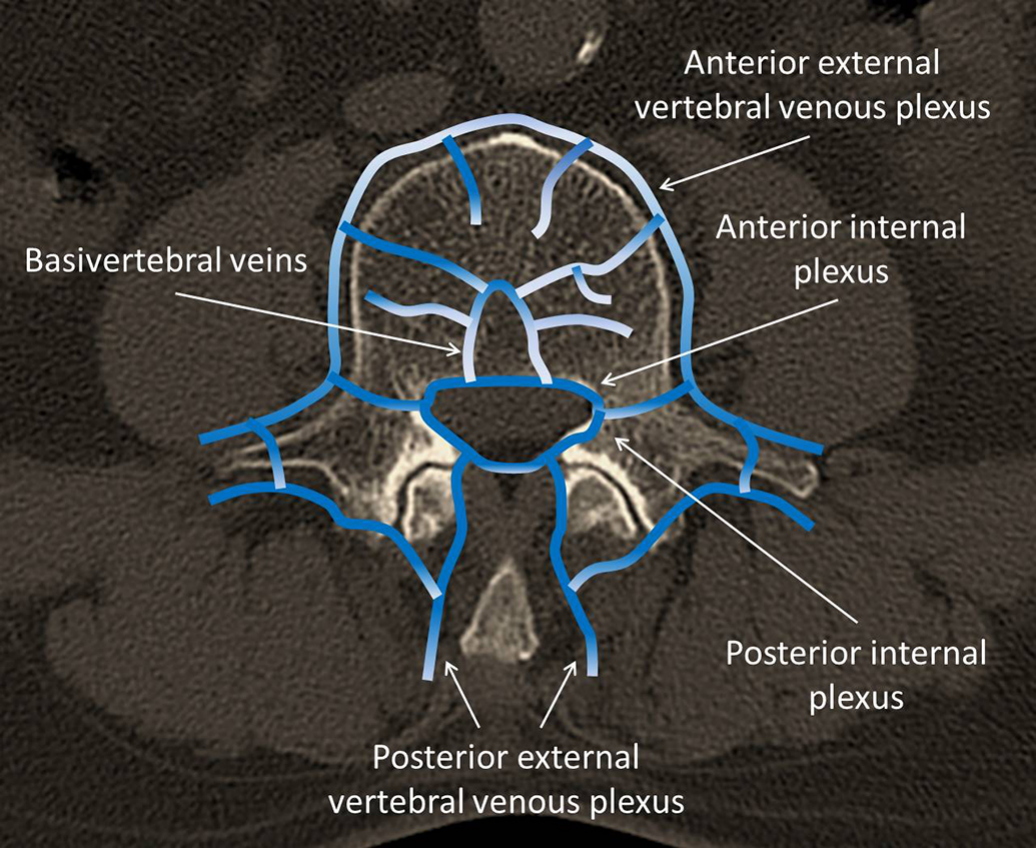
Schema of the vertebral venous plexuses.

Vertebral venous plexuses exist around the bodies of the vertebrae, namely, the anterior external vertebral plexuses and the posterior vertebral venous plexuses. The anterior external vertebral plexuses lie anterior and lateral to the vertebral bodies and communicate with the intervertebral and basivertebral veins. Intervertebral veins receive blood from the veins of the spinal cord and internal vertebral venous plexuses. The posterior vertebral external plexuses lie partly posterior to the surfaces of the vertebral arches and their processes (Figure 3) ^(24)^.

In the aforementioned three cases, no clinical symptoms were observed; however, intraoperative X-ray and postoperative CT revealed cement embolisms into the anterior external plexuses on the lateral vertebral body. Probably, the cement flowed from the basivertebral veins in the vertebral body to the anterior external plexuses. The cement may flow from the anterior external plexuses to the hemiazygos vein and the azygos vein and eventually from the inferior vena cava to the pulmonary artery, causing PCE. Normally, the pressure to which the balloon is inflated is approximately 100 psi; however, in all those three cases, the pressure had increased to 145 psi or more from the beginning or in the middle of the procedure. In fact, the BEP in the group with cement embolism was significantly higher than that in the group without cement embolism. Accordingly, we consider it important to pay close attention to cement pressure from about 145 psi to ensure that no embolization enters into the perivertebral veins.

Furthermore, if the viscosity of the bone cement is low, it is naturally more likely that it would enter into the perivertebral veins. It is generally deduced that the optimum viscosity for injection is such that when the cement is stretched between the thumb and index finger, it hardly draws threads. It depends on the temperature of the operation room and the timing of the removal of the cement components from the refrigerator. However, in general, it is necessary to wait for at least 5-10 min after mixing the bone cement to start injection after the cement has hardened to some extent.

To perform BKP more safely, it must be ensured that the first bone access needle crosses the posterior wall of the vertebral body, paying attention to avoid nerve damage. At the time of balloon expansion, care must be taken when the balloon hits the upper and lower end plates of the vertebral body so that the sphere does not become too flattened. Also, since the volume of the vertebral body is different at each level, the amount of cement required depends on the size of the fractured vertebral body. The average amount of cement used this time was 5.9 mL, and when using more cement than this, the X-ray fluoroscopy image must be checked as appropriate, and attention should be paid to whether the cement is leaking outside the vertebral body or invading the blood vessels.

In addition, we consider it important to avoid cement inflow into any blood vessel, considering that intraoperative X-ray imaging shows the expansion of the cement mass into the bone marrow cavity as if extending a tentacle from the periphery, indicating a high cement injection pressure.

As for the limitations in this study, the number of cases this time was not so large, and it may be necessary to repeat it and increase the sample size. Also, as aforementioned, while there are many reports on the effectiveness of BKP, the use of BKP for OVCFs seems to be controversial ^(25)^. In the future, more appropriate and careful BKP adaptation to OVCFs based on reliable data is required.

## Conclusion

While there is no doubt that BKP will significantly contribute to the treatment of OVCFs, we suggest that great care should be taken in determining the surgical indications for it. Furthermore, although BKP invasion is small, care must be taken to ensure that it can be performed safely and with the correct procedural factors, especially the BEP. That is to say, we consider it important to pay close attention to cement pressure from about 145 psi to ensure that no embolization enters into the perivertebral veins. Careful attention should be paid to the prevention of complications and appropriate management of any complications.

## Article Information

### Conflicts of Interest

None

### Author Contributions

Y. T., S. M., and M. M. performed operations. Y. T. wrote the manuscript. T. N. and T. M. contributed to the writing of the manuscript.

### Approval by Institutional Review Board (IRB)

Approval code issued by the institutional review board (IRB): No, 11

The name of the institution(s) that granted the approval: Musashino General Hospital

### Ethical Approval

All procedures were in accordance with the ethical standards of our hospital and with the 1964 Declaration of Helsinki and its later amendments or comparable ethical standards.

### Informed Consent

Informed consent was obtained from all individual participants included in the study.
